# Trends in HbA1c and other biochemical outcomes of individuals with newly diagnosed type 1 diabetes

**DOI:** 10.1007/s11845-020-02434-w

**Published:** 2020-11-24

**Authors:** A. O’Carroll-Lolait, A. Urwin, I. Doughty, J. Schofield, H Thabit, L. Leelarathna

**Affiliations:** 1 School of Medicine, Faculty of Biology, Medicine and Health, University of Manchester, Manchester, UK; 2 Manchester Diabetes Centre, Manchester University NHS Foundation Trust, Manchester Academic Health Science Centre, Manchester, UK; 3 Royal Manchester Children’s Hospital, Manchester University NHS foundation Trust, Manchester, UK; 4 Division of Diabetes, Endocrinology and Gastroenterology, Faculty of Biology, Medicine and Health, University of Manchester, Manchester, UK

**Keywords:** Cardiovascular risk factors, Complications, HbA1c, Type 1 diabetes

## Abstract

**Background:**

There is limited data on glycaemic control and cardiovascular risk factor management in newly diagnosed individuals with type 1 diabetes in the first 2 years.

**Methods:**

Retrospective, single centre study from the North West of England, newly diagnosed with type 1 diabetes between 2014 and 2018 (*n* = 58). HbA1c, blood pressure, lipids and body mass index (BMI) data were collected from electronic patient records from the time of diagnosis until the end of 2 years, stratified by age 16–24 years or ≥ 25 years at presentation.

**Results:**

For those aged 16–24 years (*n* = 31), median (IQR), HbA1c improved at 6 months from 83 (63–93) to 51.5 (46–75) mmol/mol (*p* = 0.001) and remained stable 6–24 months. For those ≥ 25 years (*n* = 27), HbA1c declined from 91 (70–107) to 65 (50–89) mmol/mol, (*p* < 0.01) at 6 months and declined further to 52 mmol/mol (44–70) at 24 months. At 24 months, 27.8% of all individuals had an HbA1c ≥ 69 mmol/mol. Approximately, a third met LDL (< 2 mmol/L) and total cholesterol (< 4 mmol/L) targets. A total of 58.6% of individuals were overweight/obese (BMI > 25 kg/m^2^) at 24 months compared to 45.8% at baseline. There were no significant blood pressure changes during the follow-up.

**Conclusions:**

In both age groups, significant improvement of HbA1c occurred within the first 6 months of diagnosis with no statistical difference between the two groups at any of the time points up to 24 months. Despite significant improvements in HbA1c, majority had levels > 53 mmol/mol at 24 months. Alongside the high incidence of obesity and dyslipidaemia, our data support the need for further intensification of therapy from diagnosis of type 1 diabetes.

## Introduction

Diabetes is one of the most common chronic medical conditions in Europe. Relative to the adult population, the incidence of new onset type 1 diabetes (T1DM) is higher in adolescents but the prevalence is increasing in all age groups.[1]. The classic symptoms of polydipsia, polyuria, tiredness and weight loss, along with overt hyperglycaemia, are more commonly seen in children, with the presentation in adults often more variable.[2] Great effort is required from both patients and carers to lower HbA1c levels to the recommended targets, due to the need to maintain lifelong insulin administration, regular blood glucose self-monitoring and stringent lifestyle management to achieve optimal glycaemic control. Healthcare providers use HbA1c measurements to monitor patients’ long-term glycaemic control, but target HbA1c levels differ between countries and ages. The National Institute for Health and Care Excellence (NICE) in the UK recommends a target HbA1c of < 48 mmol/mol for both children and adults to minimise the risk of developing complications.[3] Whereas in the USA, the American Diabetes Association (ADA) recommends a target < 58 mmol/mol for children and < 53 mmol/mol for adults.[4] Individuals with diabetes are at higher risk of cardiovascular disease (CVD). This risk can be estimated through assessing multiple biochemical factors. NICE no longer specify exact targets for lipid measurements for people with T1DM, however to reduce the risk of CVD, targets of < 4 mmol/L for total cholesterol (TC), < 2 mmol/L for LDL, ≤ 1.7 mmol/L for triglycerides and either ≥ 1.0 mmol/L (men) or ≥ 1.3 mmol/L (women) for HDL, have been identified by Diabetes UK as a guide for people with T1DM.[5] The combination of poor glycaemic control with dyslipidaemia and obesity predisposes individuals with T1DM to develop micro- and macrovascular complications.

Evaluating the actual physical, social and economic burdens of T1DM is challenging. [1]. However, it has been well documented that achieving optimal glycaemic control is important to reduce the incidence of developing complications associated with significant morbidity and mortality.[6] Complications can be categorised into microvascular (retinopathy, nephropathy or neuropathy) or macrovascular (CVD, cerebrovascular disease and peripheral vascular disease).[7] Results from the Diabetes Control and Complications Trial (DCCT) and its long-term follow-up, the Epidemiology of Diabetes Interventions and Complications (EDIC) study have clearly demonstrated that hyperglycaemia has a causal role in the development and progression of both microvascular and macrovascular complications.[8, 9] CVD is more prevalent amongst those with T1DM than the nondiabetic population, with a 10-fold increase of CVD seen in those with T1DM than without.[10] There are multiple identifiable risk factors associated with CVD, such as HbA1c, blood pressure (BP), obesity and lipids, that can be monitored and managed in those with T1DM to reduce this risk.[6] The purpose of this study was to look at progression of HbA1c levels, and cardiovascular risk factors during the first 2 years of T1DM diagnosis.

## Materials and methods

This was a retrospective, observational, single-centre, service evaluation from the North West of England. Results were included for those diagnosed with T1DM between 2014 and 2018, and who have a minimum of 12 months of subsequent electronic clinic records data.

As this was a service evaluation, no ethical approval was required. HbA1c values were obtained at 6 monthly intervals from presentation throughout the first 2 years. Data on BP, lipids, and body mass index (BMI) were collected at presentation, and 12 and 24 months post-diagnosis, as these are the time points at which patients undergo more extensive evaluation.

Glycaemic control was assessed using targets outlined by NICE. [3] HbA1c levels of ≤ 48 (≤ 6.5%), ≤ 58 (≤ 7.5%) and ≥ 69 (≥ 8.5%) mmol/mol were used as targets to assess glycaemic control amongst our cohort. BP control was assessed using NICE-recommended targets of systolic BP < 135 mmHg and diastolic BP < 85 mmHg.[3] BP measurements were typically recorded using a single sitting reading. If the measurement was raised, a second sitting reading after 5 min was performed and the lower of the two values was used. BMI measurements were categorised as underweight (< 18 kg/m^2^), normal (18–25 kg/m^2^), overweight (25–30 kg/m^2^) and obese (> 30 kg/m^2^). Target lipid levels were defined as TC < 4 mmol/L, LDL < 2.0 mmol/L, HDL > 1.0 mmol/L (men) or > 1.3 mmol/L (women) and triglycerides < 1.7 mmol/L. A less aggressive LDL target of < 2.6 mmol/l, consistent with ADA, ISPAD and ESC/EASD recommendations [2, 11] was used for secondary analysis.

The cohort was stratified by age at presentation into those aged 16−24 years and those aged ≥ 25 years at diagnosis. This allowed comparisons to be made and analysed between the two groups.

We used the IBM SPSS version 25 for statistical analysis. Data were mostly non-normally distributed so we have shown median (IQR) and used non-parametric tests for comparison of two groups.

## Results

Between January 2014 and April 2018, 58 patients between the ages of 16 and 54 years were diagnosed with T1DM at Manchester Royal Infirmary. Participants were divided into young adult (16–24 years old, *n* = 31) and adult (25 years and above, *n* = 27) age groups. Baseline characteristics for the whole cohort are shown in Table 1.

**Table 1:** Baseline characteristics

Characteristic	Data	*N*(%)
*N*	58	
Females	34 (59%)	
Age at diagnosis (year)	24 (20, 32)	
Aged 16–24 years*	31 (53%)	
HbA1c (mmol/mol)	86 (66, 95)	31 (53)
TCa (mmol/L)	4.20 (3.75, 4.90)	53 (91)
HDL-Cb (mmol/L)	1.57 (1.27, 1.83)	53 (91)
LDL-C^c^ (mmol/L)	2.21 (1.62, 2.78)	50 (86)
Triglycerides (mmol/L)	1.00 (0.70, 1.50)	53 (91)
BMI^d^ (kg/m^2^)	24.2 (22.1, 27.1)	35 (60)

Data are *N* (%) or median (IQR) unless stated

*N* (%), number of patients with data (percentage of total cohort)

^a^*TC*, total cholesterol

^b^*HDL-C*, high-density lipoprotein cholesterol

^c^*LDL-C*, low-density lipoprotein cholesterol

^d^*BMI*, body mass index

*No. of patients aged 16–24 at time of diagnosis

### Glycaemic control (Table 2)

HbA1c measurement availability at diagnosis, 6, 12, 18 and 24 months was 53%, 79%, 77%, 63% and 62%. Table 2 shows median HbA1c levels for the whole cohort and stratified by age group at 6 monthly intervals from diagnosis to 2 years post-diagnosis, and the proportion (%) of patients reaching HbA1c ≤ 48 and ≤ 58 mmol/mol.

**Table 2:** Glycaemic control covering the first 2 years of diagnosis

	Diagnosis	6 months	12 months	18 months	24 months
Whole cohort					
HbA1c (mmol/mol)	86 (66, 95)	58.8 (48, 76)	52.5 (45, 66)	55 (48, 70)	52 (46, 70)
*N* (%)	31 (53)	46 (80)	45 (78)	37 (64)	36 (62)
16–24 years					
HbA1c (mmol/mol)	83 (63, 93)	51.5 (46, 75)	51 (45, 64)	59 (48, 63)	53 (49, 70)
≤ 48(%)	0	36.4	34.8	23.5	23.5
≤ 58 (%)	12.5	59.1	65.2	47.1	70.6
≥ 69 (%)	68.8	19.3	6.5	17.6	29.4
≥ 25 years					
HbA1c (mmol/mol)	91 (70, 107)	65 (50, 89)	53.5 (44, 72)	54 (47, 72)	52 (44, 70)
≤ 48(%)	0	16.7	36.4	35.0	36.8
≤ 58 (%)	0	41.7	59.1	55.0	63.2
≥ 69(%)	80.0	41.7	31.8	35.0	26.3
*p* value	0.143	0.099	0.307	0.647	0.612

Data are median (IQR) and % unless stated

*N* (%), number of patients with data (percentage of total cohort)

*p* value is the comparison between the two cohorts at each time point (Mann-Whitney *U* test)

For the whole cohort, we noted significant improvement in HbA1c between diagnosis and 24 months, 86 (66, 95) to 52 (46, 70) mmol/mol, *p* = 0.001. This improvement predominantly occurred between the diagnosis and 6 months, 86 (66, 95) to 58.8 (48, 76) mmol/mol, *p* < 0.001. Median HbA1c at diagnosis was higher in the older cohort than the younger (91 vs 83 mmol/mol) but did not reach statistical significance (*p* = 0.143). (Table 2) Similarly, the older group had higher median HbA1c levels during the initial 12 months of the study period compared to the younger group, without statistical significance. At 24 months, both groups recorded similar median HbA1c results.

In the younger cohort, the proportion of patients achieving a HbA1c ≤ 48 mmol/mol decreased from 6 to 24 months post-diagnosis (36.4 to 23.5%) but the proportion achieving a HbA1c ≤ 58 mmol/mol increased (59.1 to 70.6%), whilst in the older cohort, the proportion of patients achieving both of these targets rose with 36.8% achieving a HbA1c ≤ 48 mmol/mol at 24 months (a rise from 16.7% at 6 months) and 63.2% achieving a HbA1c ≤ 58 mmol/mol (a rise from 41.7% at 6 months). At 24 months, 27.8% of all individuals had a HbA1c ≥ 69 mmol/mol, with 29.4% of those aged 16–24 and 26.3% of those ≥ 25 within this range.

### Cardiovascular risk factor levels

Data availability at 24 months for SBP, DBP, TC, LDL, HDL, triglycerides and BMI were 76%, 76%, 78%, 74%, 78%, 78% and 84%, respectively. In the older group, hypercholesterolaemia (TC > 4 mmol/L) was found in 62.5% 24 months post-diagnosis, with 47.8% having an LDL cholesterol > 2 mmol/L and 30.4% failing to reach the recommended LDL target of < 2.6 mmol/L.[2, 11] All of those aged ≥ 25 years achieved satisfactory HDL cholesterol levels (> 1.0 mmol/L for men and > 1.3 mmol/L for women) but hypertriglyceridaemia (< 1.7 mmol/L) was observed in 25% at 24 months post-diagnosis.

A significant percentage, 50% (9 out of 18), of the older cohort were overweight (BMI > 25) or obese (BMI > 30 kg/m2) at time of diagnosis, which increased to 63.7% (14 out of 22) at 24 months post-diagnosis. Only 31.8% (7 out of 22) had a BMI within the ideal range (18 to 25 kg/m^2^) 24 months after diagnosis. BP levels were within range throughout the study period, with recommended systolic and diastolic targets met by the majority (81.8% for both) by study end.

The 16–24 age group showed similar results at 24 months, with hypercholesterolaemia found in 76.2% of individuals (45% had LDL > 2.6 mmol/L) whilst high proportions achieved HDL and BP targets (95.2, 81.8 (systolic) and 100% (diastolic), respectively). Furthermore, overweight and obesity were common with 52.6% (10 out of 19) having a BMI > 25 kg/m^2^ at 24 months, an increase from 41.2% (7 out of 17) at diagnosis, with less than half achieving a BMI within the ideal range.

### Participation in structured education (DAFNE course)

Of the total cohort, 11 (19%) individuals had undertaken the Dose Adjustment for Normal Eating (DAFNE) course. HbA1c at 24 months in those who participated in the DAFNE course was not significantly different to those who did not do the DAFNE course. Amongst DAFNE attenders, the pre-course mean HbA1c was 58.8 mmol/mol whilst post-course mean HbA1c was 59.0 mmol/mol (no statistically significant difference).

## Discussion

### Main findings

In this study, we describe the changes in HbA1c and other cardiovascular risk factors in a cohort of patients with newly diagnosed type 1 diabetes presenting between 2014 and 2018 at a single centre followed up for 24 months. We observed significant improvements in the HbA1c within 6 months of the diagnosis which remained largely stable in the 6- to 24-month period. The percentage of people with HbA1c ≤ 58 mmol/mol was highest at 24 months for both age groups, but the percentage of people with HbA1c ≤ 48 mmol/mol was highest in 16–24 cohorts at 6 months. A relatively large proportion of both cohorts still recorded HbA1c levels above 69 mmol/mol, 24 months after diagnosis, which is concerning given the well-known relationship between hyperglycaemia and development of long-term complications.**[**8, 9] Also of concern is the increasing prevalence of overweight and obesity within the cohort covered in this study, due to the adverse CVD risk outcomes associated with elevated BMI. Over half of the included population had a BMI > 25 kg/m^2^ 24 months after diagnosis.

### Comparison with published data

Our BMI data are comparable with findings from the Health Survey for England conducted in 2018 in which 63% of all adults (> 16) were overweight or obese. [12] One-third of the entire cohort in this study had hypercholesterolaemia (TC > 5 mmol/L) 24 months post-diagnosis, fewer than the 47% of adults reported to have hypercholesterolaemia in the health survey. [12]. This remains a cause for concern given the strong association between age at onset of type 1 diabetes and excess cardiovascular disease [13] and evidence that lipid-lowering therapy is associated with a significant cardiovascular disease risk reduction amongst individuals with type 1 diabetes [14]. Adoption of the ADA and ISPAD LDL target of < 2.6 mmol/L for people with type 1 diabetes would identify 37.2% of this cohort as being ‘above target’.[15] Attainment of target blood pressure measurements remained high within both cohorts throughout the study period.

The UK National Diabetes Audit 2019–2020 (*n* = 245,565) reported that 31.8% of patients with type 1 diabetes of all ages achieved a HbA1c ≤ 58 mmol/mol and 10.6% met the target of ≤ 48 mmol/mol [16]. Although not directly comparable due to different duration of diabetes in these two cohorts, in our cohort, 70.6% of those 16–24 and 63.2% of those ≥ 25 years achieved a HbA1c ≤ 58 mmol/mol, and 23.5 and 36.8% (16–24 and ≥ 25, respectively) meet the ≤ 48 mmol/mol target.[16] The national audit also reported 73.1% of patients meeting the BP target of 140/80 mmHg, with a higher proportion of patients in this study (93%) achieving the same target.

Although large observational data sets are available, none have focussed solely on newly diagnosed patients. Internationally, the Swedish National Diabetes Register (NDR) publishes reports on national adult diabetes care.[17, 18] Their study reported a mean HbA1c of 62.8 mmol/mol for patients with T1DM seen in specialist clinics in 2015 (*n* = 33,287). They demonstrated that increasing proportions of patients were achieving treatment targets for HbA1c over the study period. Other biochemical parameters associated with the development of complications were also evaluated. Mean BP decreased throughout the study, and the proportion meeting the 140/85 mmHg target stood at 78.6% at the end of 4 years. This study did not identify the results for newly diagnosed patients, so a direct comparison with our results could not be made.

Furthermore, the USA Type 1 Exchange Registry evaluated the HbA1c data of patients between 2016 and 2018 (*n* = 22,697).[19] The ADA HbA1c goals for children (< 58 mmol/mol) and adults (< 53 mmol/mol) were being met by 17% and 21% of patients, respectively, considerably lower than the proportions meeting HbA1c targets in our study; however, the US study also did not identify newly diagnosed patients, so a direct comparison of results cannot be made.

Any differences in HbA1c values in relation to enrolment on a DAFNE course were also analysed. Our results highlight no statistical difference in the HbA1c values pre- and post-enrolment on the course. This result is not surprising, as all participants were at early stages of their diagnosis.

### Strengths and limitations

To our knowledge, this is the first study in the UK to report the evolution in HbA1c and cardiovascular risk factors in the first 2 years of diagnosis for both young adult and adult populations. Heald et al. published a study focusing on the intervention of diabetes specialist nurse services and the subsequent effects on HbA1c levels over an 18-month follow-up period from diagnosis. [20] The authors reported similar findings with significant initial reductions in HbA1c, falling from 99 mmol/mol at diagnosis to 49 mmol/mol at 6 months (*p* < 0.001), followed by no further improvement during the study period. Higher percentages of patients achieved HbA1c treatment targets at the end of the study, 84.6% achieved a HbA1c < 58 mmol/mol and 61.5% met a target of < 48 mmol/mol. However, the study was limited by its small sample size (*n* = 15) and population (only included adults). Our study is larger (*n* = 58) and includes a wider age range of participants, providing a better estimate of the evolution of glucose control in those initial years. A key limitation is the retrospective nature of this study, including the lack of information on each parameter for all individuals at each time point. For example, only 62% had HbA1c data at 24 months. Therefore, it is possible that the percentage of individuals with HbA1c > 69 mmol/mol could be higher than 27.8%, as the HbA1c in those who have not attended clinic appointments could be higher. Although it is standard practice in our centre to perform autoantibodies measurements at diagnosis for anti-GAD and Islet cell antibodies, for the purpose of this analysis information on autoantibodies measurements were not collected. We therefore acknowledge that a minority in our cohort may have other types of diabetes.

In summary, our data show good improvements in HbA1c since diagnosis of type 1 diabetes in a cohort of adults and adolescents. Despite the initial improvements, more than a quarter of the population had very poor glucose control (HbA1c ≥ 69 mmol/mol) at 2 years. It will be very important to identify these individuals as soon as possible and institute additional measures to improve glycaemia in this group. In addition, a significant proportion of the cohort was overweight or obese at diagnosis with further weight gain during the first 2 years after diagnosis and associated high cholesterol levels. The 2019 ESC/EASD Guidelines have adopted new language around risk, with younger patients with short duration of diabetes and no other recognised risk factors still considered to be at moderate risk [21]. Our data calls for more aggressive management of glycaemic targets and cardiovascular risk factors in newly diagnosed patients with type 1 diabetes thereby reducing the risk of developing long-term micro- and macrovascular complications.

## Data Availability

Data was retrieved from electronic patient records.

## Abbreviations

BMI: body mass index
HDL: high-density lipoprotein
LDL: low-density lipoprotein
TC: total cholesterol
BP: blood pressure
T1DM: type 1 diabetes mellitus
HbA1c: haemoglobin A1c
